# Accumulation of ALDH1-positive cells after neoadjuvant chemotherapy predicts treatment resistance and prognosticates poor outcome in ovarian cancer

**DOI:** 10.18632/oncotarget.4103

**Published:** 2015-05-11

**Authors:** Tiyasha H. Ayub, Mignon-Denise Keyver-Paik, Manuel Debald, Babak Rostamzadeh, Thore Thiesler, Lars Schröder, Winfried Barchet, Alina Abramian, Christina Kaiser, Glen Kristiansen, Walther Kuhn, Kirsten Kübler

**Affiliations:** ^1^ Department of Obstetrics and Gynecology, Center for Integrated Oncology, University of Bonn, Sigmund-Freud-Strasse, Bonn, Germany; ^2^ Institute of Pathology, Center for Integrated Oncology, Sigmund-Freud-Strasse, Bonn, Germany; ^3^ Institute of Clinical Chemistry and Clinical Pharmacology, Center for Integrated Oncology, Sigmund-Freud-Strasse, Bonn, Germany

**Keywords:** ovarian cancer, predictive marker, prognostic marker, ALDH1, cancer stem-like cell

## Abstract

Although ovarian cancer is a highly chemosensitive disease, it is only infrequently cured. One of the major reasons lies in the presence of drug-resistant cancer stem-like cells, sufficient to fuel recurrence. We phenotyped cancer stem-like cells by flow cytometry and immunohistochemistry in 55 matched samples before and after taxane/platinum-based neoadjuvant chemotherapy. All used markers of stemness (ALDH1, CD24, CD117, CD133) isolated low frequencies of malignant cells. ALDH1 was the most valuable marker for tracking stemness *in vivo*. The enrichment of ALDH1 expression after treatment was associated with a poor response to chemotherapy, with platinum resistance and independently prognosticated unfavorable outcome. Our results suggest that increased ALDH1 expression after treatment identifies patients with aggressive tumor phenotypes.

## INTRODUCTION

Despite being the second most common malignancy of the female genital tract in developed countries, epithelial ovarian cancer (EOC) is the leading cause of death from all gynecologic tumors and the fifth most lethal type of cancer in women. Over the past two decades the median survival has improved due to more aggressive surgical techniques [1] and optimized combinations of cytotoxic drugs [2, 3] but the long-term cure rate remains as low as 30%. Thus, with an age-standardized mortality rate of 5.1/100.000 person-years EOC has significant implications for public health and social costs [4]. A major reason for this high death toll is the development of chemoresistance in the course of the disease and especially platinum-resistant disease is uniformly fatal. Even though a first complete clinical and pathological remission is achieved in at least 50% of these women with cytoreductive surgery and taxane/platinum-based chemotherapy, more than 70% of patients experience relapse and eventually succumb to the disease.

Different factors have been shown to contribute to treatment failure in cancer. Among these, cancer stem cells (CSCs), cancer-/tumor-initiating cells (CICs, TICs; from this point onward referred to as CSCs) or cancer stem-like cells (CSLCs; from this point onward referred to as CSCs) came into the focus of interest during the last decade. Solid tumors are known to be composed of different cell populations, of which one, in contrast to cancer cells with a limited proliferative potential, is able to self-renew and to maintain the tumor [5]. The existence of these slow-dividing immortal cells explain why current anti-cancer agents are effective in reducing the tumor mass but by selecting for oncogenic resistance only infrequently cure the patient [6, 7]. CSCs rely on a network of highly conserved embryonic signaling pathways, including Notch, Hedgehog and Wnt, which are vital for self-propagation. CSCs also exhibit resistance to treatment with genotoxic agents mainly due to active DNA repair machinery, the expression of ATB-binding cassette (ABC) drug transporters and resistance to apoptosis [8].

CSCs have been isolated from multiple cancer entities and correlated to therapeutic resistance and poor prognosis [9]. Depending on the tumor context various markers have been suggested to be useful to identify CSCs but a single universal indicator has not been discovered yet. In fact, its existence is questionable given CSC phenotype variations both among tumors and within a tumor. Also for ovarian CSCs, a specific phenotype has not been determined so far. Given the heterogeneity of the tumor bulk in advanced stages it even seems unlikely that one universal marker would be sufficient to describe all ovarian CSCs. Candidate markers include aldehyde dehydrogenase 1 (ALDH1) [10-16], mucin-type glycoprotein CD24 [17, 18], proto-oncoprotein CD117 or c-Kit [19-21] and transmembrane glycoprotein CD133 [22].

Here, we used matched pairs of chemotherapy-naïve tissue specimens taken at the time of diagnosis and taxane/platinum-treated samples taken during radical interval debulking surgery (IDS) to characterize residual tumor cells that have survived chemotherapy using several well-characterized markers of stemness. These CSC populations were then tested as predictors of chemotherapeutic response and prognosticators of recurrence-free and overall survival. The goal of our study was to provide the rationale for the improvement of CSC-focused anticancer strategies able to abrogate chemoresistance and thus to improve outcome.

## RESULTS

### CSCs are present in ascites of therapy-naïve EOC patients as detected by flow cytometry

**Figure 1:**
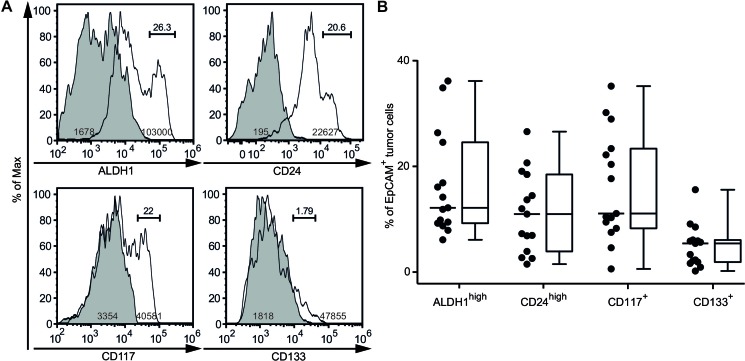
Identification of ovarian CSCs by flow cytometry **A.** Cytoplasmic expression of ALDH1 and cell surface expression of CD24, CD117 and CD133 on ascites-derived EpCAM^+^ cells. Staining was determined by flow cytometry in 15 therapy-naïve patients with EOC; representative data from one patient are shown. Histograms show control (shaded) and specific (open) staining; numbers within peaks refer to geometric mean fluorescence intensity (MFI) of CSCs; numbers above bars indicate the percentage of CSCs. **B.** Frequencies of ascites-derived EpCAM^+^ cells expressing ALDH1, CD24, CD117 and CD133. Data were analyzed as in A. Dots represent individual tumors; bars indicate the median; box plots summarize the median, 25^th^ and 75^th^ percentiles; whiskers indicate minimum and maximum values.

Advanced EOC is typically associated with ascites, which is considered an adequate model for the tumor and its microenvironment. We have previously identified epithelial cell adhesion molecule (EpCAM)^+^ EOC cells as the second largest ascitic cellular population in therapy-naïve women [23]. Based on this finding, we determined the frequency of ascitic CSCs in the subset of EpCAM^+^ EOC cells using previously reported markers of tumorigenicity [24]. Two of these antigens (ALDH1, CD24) were distributed in a nearly ubiquitous fashion on cancer cells (Figure 1A). However, CSCs could be differentiated from non-tumorigenic cells by their high staining intensity. Accordingly, EOC cells were split into high versus low ALDH1 and CD24 expression groups. Other CSC phenotypes (CD117, CD133) were identified by positive labeling in contrast to absent expression in non-tumorigenic cells. The percentage of CSCs in ascites varied between 0.12% and 36.1% (Figure 1B, Table 1). We found that the quantities of ALDH1^high^, CD24^high^ and CD117^+^ CSCs were of similar magnitude while the proportion of CD133^+^ CSCs was lower suggesting phenotypic heterogeneity. Additionally, we performed a comparative analysis of stemness antigen expression in malignant and benign tissue and found that CSC-related molecules are also distributed on selected normal cells (Supplementary Figure S1). However, antigen signals on immunocytes were always lower than those of CSCs.

**Table 1:** Clinicopathological patient characteristics

Variable	Value [median ± MAD (range)]
Age (yrs)		55 ± 8.9 (34 - 79)
Time from laparoscopy to CRS (ds)		63 ± 11.86 (39 - 145)
Ascitic CSCs (% of tumor cells)	EpCAM^+^ALDH1^high^	12.1 ± 5.78 (6.02 - 36.1)
	EpCAM^+^CD24^high^	10.9 ± 10.47 (1.43 - 26.5)
	EpCAM^+^CD117^+^	11 ± 9.79 (0.54 - 35.1)
	EpCAM^+^CD133^+^	5.34 ± 4.64 (0.12 - 15.5)
Pre-chemotherapeutic CSCs (IRS)	ALDH1	4 ± 2.97 (0 - 9)
	CD24	6 ± 2.97 (0 - 12)
	CD117	0 ± 0 (0 - 4)
	CD133	0 ± 0 (0 - 4)
Post-chemotherapeutic CSCs (IRS)	ALDH1	8 ± 7.90 (0 - 36)
	CD24	8 ± 11.86 (0 - 48)
	CD117	0 ± 0 (0 - 24)
	CD133	0 ± 0 (0 - 8)
**Variable**		**Value** [median (95% CI)]
Follow-up time (ms)		44.81 (17.01- 72.61)
**Variable**		**Value** [n (%)]
Stage	IIIC	45 (81.8)
	IV	10 (18.2)
Grade	2	14 (25.5)
	3	40 (72.7)
	ND	1 (1.8)
Lymph node metastasis	Absent	23 (41.8)
	Present	30 (54.5)
	ND	2 (3.6)
Pre-chemotherapeutic site of tissue origin	Peritoneum	47 (85.5)
	Ovary	7 (12.7)
	Greater omentum	1 (1.8)
Post-chemotherapeutic site of tissue origin	Peritoneum	1 (1.8)
	Ovary	54 (98.2)
Chemotherapeutic cycles at 3-wkly intervals	2 neoadjuvant + 4 adjuvant	36 (65.5)*
	3 neoadjuvant + 3 adjuvant	19 (34.5)
Chemotherapeutic agents	C	2 (3.6)
	C + P	33 (60)**
	C + D	20 (36.4)
Histopathologic response to NAC	Non-responder	33 (60)
	Responder	22 (40)
RCB after cytoreductive surgery	< 1cm [NRD]	48 (87.3) [27 (49.1)]
	> 1cm	7 (12.7)
Disease status	No evidence of disease	7 (12.7)
	Recurrent disease	48 (87.3)
Outcome	Alive	20 (36.4)
	Dead	35 (63.6)

MAD, median absolute deviation; IRS, immunoreactivity score; CI, confidence interval;

CRS, cytoreductive surgery; yrs, years; ms, months; wkly, weekly; ds, days; ND, not determined;

RCB, residual cancer burden; C, carboplatin AUC5; P, paclitaxel (175mg/m^2^); D, docetaxel (75mg/m^2^);

NAC, neoadjuvant chemotherapy; NRD, no macroscopic residual disease.

* includes the case of one missed adjuvant cycle due to pelvic abscess.

** includes one case that switched over to docetaxel due to neurotoxic side-effects.

### CSCs are present in solid therapy-naïve EOC specimens as detected by immunohistochemstry

**Figure 2:**
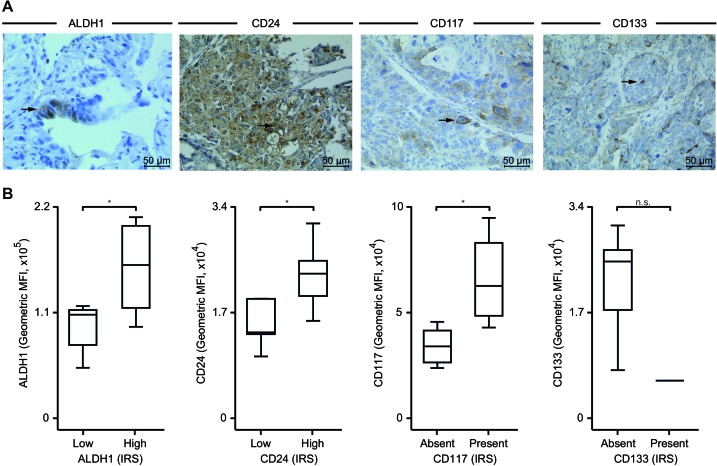
Immunohistochemistry-based quantification of CSCs **A**. Representative images of ALDH1, CD24, CD117 and CD133 expression in EOC visualized by immunohistochemistry (brown, arrow); hematoxylin (blue) was used for nuclear staining (bright field image, 400x magnification). **B.** Expression of tumoral ALDH1, CD24, CD117 and CD133 was assessed by immunohistochemistry in pre-chemotherapeutic tissue samples and gave rise to an immunoreactivity score (IRS, see Methods for details); samples were divided by the median into low and high or absent and present expression groups. Additionally, stemness antigens were analyzed by flow cytometry as in Figure 1A; data are given as geometric MFI values. Box plots summarize the median, 25th and 75th percentiles, the whiskers and outliers (*, *p* < 0.05; n.s., not significant).

We screened solid tumors for the existence of ovarian CSCs to provide a basis for the evaluation of the tumoral response to chemotherapy. In accordance to our flow cytometric data, the immunohistochemical approach identified a small percentage of ALDH1^high^, CD24^high^, CD117^+^ and CD133^+^ EOC cells (Figure 2A, Table 1). The analyses of solid tumors and ascites gave similar results for the quantification of ALDH1^high^ (*p* < 0.05), CD24^high^ (*p* < 0.04) and CD117^+^ (*p* < 0.02) CSCs indicating the validity of both methods and the presence of ovarian CSCs (Figure 2B). Only with regard to CD133^+^ the two methods detected different frequencies presumably due to low antigen expression.

### CSC frequency in response to neoadjuvant chemotherapy

**Figure 3:**
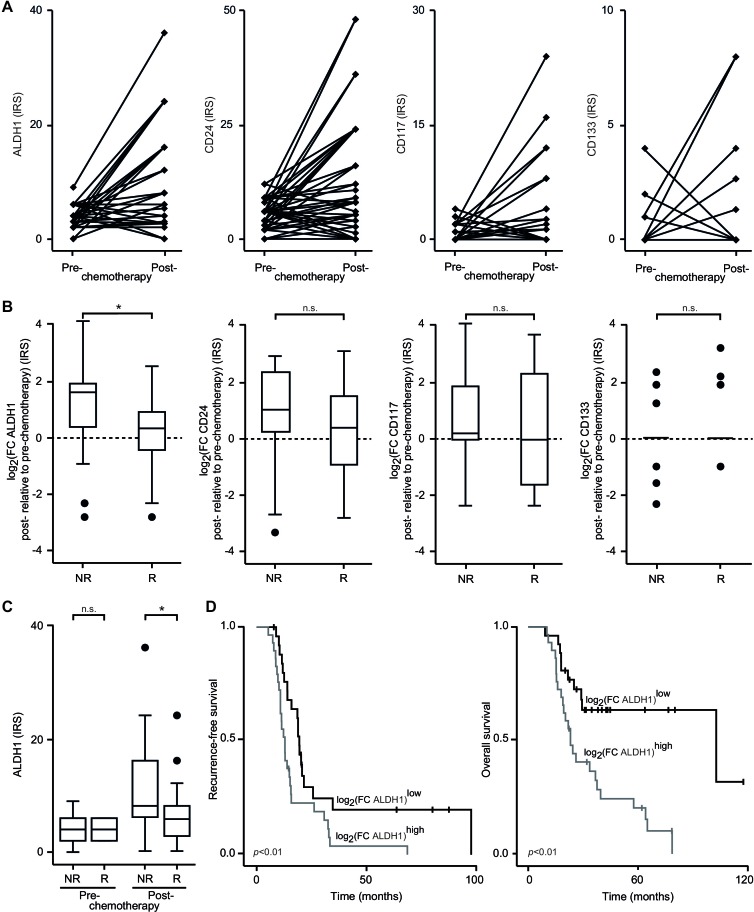
Frequency of CSCs during the course of the disease **A.** Expression of ALDH1, CD24, CD117 and CD133 was determined in cancerous tissue before and after neoadjuvant chemotherapy (NAC) by immunohistochemistry as in Figure 2B; the staining of each individual tumor is displayed; data of the same patient are connected by lines. **B.** Expression of ALDH1, CD24, CD117 and CD133 was assessed by immunohistochemistry as in Figure 2B; fold changes (FC) in expression levels were calculated as the ratio of the immunoreactivity scores in pre- to these in post-NAC tissue; classification in non-responder (NR) and responder (R) was undertaken according to the degree of histopathological tumor regression. Dashed lines indicate no change of CSC frequency; box plots summarize the median, 25th and 75th percentiles, the whiskers and outliers. **C**. Expression of ALDH1 was assessed by immunohistochemistry as in Figure 2B. Samples were grouped into treated and non-treated specimens and according to their degree of histopathological tumor regression. Box plots summarize the median, 25th and 75th percentiles, the whiskers and outliers. **D**. Kaplan-Meier curves of RFS and OS according to risk tier are shown. Expression of ALDH1 was determined by immunohistochemistry und used for statistics as in B; the cut-off value was defined by the median; results of the log-rank test are provided (*, *p* < 0.05; n.s., not significant).

In the next step we aimed to monitor the proliferation of CSCs during the course of treatment. The amount of CSCs before and after neoadjuvant chemotherapy (NAC) was evaluated in paired tissue specimens by immunohistochemistry (IHC). We were able to isolate restricted subpopulations of cancerous cells expressing stemness antigens in NAC-treated EOC samples (Table 1). However, when evaluating all patients together, no consistent pattern of change in response to anticancer therapy was found (Figure 3A).

### Chemoresistant disease is characterized by CSC accumulation

We then asked whether a distinct shift in the amount of CSCs might differentiate chemotherapeutic responders (Rs) from non-responders (NRs). To further test this theory we correlated the change of CSC antigen expression levels from pre- to post-treatment periods with histopathological features of chemoresistance. Indeed, malignancies that lacked signs of regression in response to NAC showed an accumulation of ALDH1^high^ CSCs (*p* < 0.02; Figure 3B); CD24^high^, CD117^+^ and CD133^+^ tumor cell proportions, however, were not informative for histopathological signs of regression. Remarkably, in non-regressing tumors after NAC the median of IHC-determined ALDH1 expression increased 3-fold; in single cases the increase was even up to 17-fold.

Further support for our data of CSC expansion in chemoresistant tumors came from the observation that ALDH1^high^ CSCs enriched in platinum-resistant disease (Table 2). Early relapse within 6 months of completing chemotherapy was found to occur more frequently in patients with elevated ALDH1^high^ CSC quantities after NAC. None of the other stemness antigens evaluated in our study was found to be associated with the tumoral response to platinum.

**Table 2:** CSC enrichment correlates with platinum resistance

Variable		Recurrence			**NED**	*p*-value*
		< 6 ms	6 - 12 ms	> 12 ms		
log_2_(FC in ALDH1)	low	3	7	10	6	< 0.03
	high	10	12	6	1	
log_2_(FC in CD24)	low	5	9	8	5	n.s.
	high	8	10	8	1	
log_2_(FC in CD117)	low	3	3	5	5	n.s.
	high	6	8	5	1	
log_2_(FC in CD133)	low	3	3	2	2	n.s.
	high	2	0	1	0	

FC, fold change; ms, months; NED, no evidence of disease; n.s., not significant.

* based on Monte Carlo simulations

We then aimed to know whether CSC counts might be already indicative of poor treatment response at initial diagnosis. Frequencies of ALDH1^high^ CSCs before and after NAC in NRs and Rs were determined. No difference in ALDH1^high^ cell proportions was found in chemotherapy-naïve women (Figure 3C). However, after NAC NRs showed increased numbers of ALDH1^high^ cells suggesting that CSCs expanded at the expense of their chemosensitive counterparts (*p* < 0.05).

Further association analysis of CSCs with clinical parameters confirmed our theory that ALDH1 expression is rather responsible for resistance to treatment than for promoting the disease. In detail, ALDH1^high^ cell populations were not associated with FIGO stage, tumor grade or lymph node metastasis (Supplementary Figure S2). Moreover, the number of cycles and the type of chemotherapeutic agents had no influence on the CSC count after NAC (data not shown).

Based on our observation that myeloid cells, which are morphologically similar to malignant cells, express ALDH1 (Supplementary Figure S1) the presence of aldehyde dehydrogenases was evaluated in the cancer-adjacent stroma of post-NAC tissue. However, no correlation of ALDH1^+^ monocytes/macrophages with histopathological tumor regression was found (data not shown) excluding a chemotherapy-mediated increase in macrophages that might have been able to support chemoresistance [25].

### The increase of CSCs after NAC is indicative of poor outcome

Having demonstrated that tumors that enrich CSCs after NAC are characterized by chemoresistance, we speculated that these malignancies exhibit a more aggressive phenotype. Consequently, CSCs were analyzed for their use as prognosticators of survival. The pre- to post chemotherapeutic change of ALDH1^high^ tumorigenic cells was able to distinguish between recurrence-free survival (RFS) and overall survival (OS) rates relating the accumulation of CSCs to an unfavorable outcome (Figure 3D). Consistent with these results, univariate Cox regression analysis identified an increase of ALDH1 expression after NAC as a risk factor of RFS and OS (Table 3). In the multivariate model for OS the ALDH1 score remained significant. Fascinatingly, the presence of ALDH1^high^ CSCs increased the risk of death by 4.18 times, a hazard ratio (HR) comparable to that of high RCB following cytoreductive surgery, which is known to be the most important prognostic factor in EOC and thus served as the gold standard.

**Table 3:** Risk factors affecting recurrence-free and overall survival

Variable	Recurrence-free survival					
	Univariate analysis			Multivariate analysis*		
	HR	95% CI	*p*-value	HR	95% CI	*p*-value
log_2_(FC in ALDH1)	2.16	1.19 - 3.91	< 0.02	1.77	0.91 - 3.42	0.09
log_2_(FC in CD24)	1.19	0.99 - 1.41	n.s.			
log_2_(FC in CD117)	1.2	1.03 - 1.4	< 0.03	1.15	0.96 - 1.38	n.s.
log_2_(FC in CD133)	0.94	0.67 - 1.32	n.s.			
RCB after cytoreductive surgery	2.41	1.05 - 5.53	< 0.04	2.87	1.23 - 6.72	< 0.02

Variable	Overall survival					
	Univariate analysis			Multivariate analysis*		
	HR	95% CI	*p*-value	HR	95% CI	*p*-value
log_2_(FC in ALDH1)	3.23	1.5 - 6.96	< 0.004	4.18	1.86 - 9.38	< 0.002
log_2_(FC in CD24)	1.22	0.98 - 1.52	n.s.			
log_2_(FC in CD117)	1.17	0.97 - 1.42	n.s.			
log_2_(FC in CD133)	0.61	0.12 - 3.22	n.s.			
RCB after cytoreductive surgery	2.95	1.19 - 7.29	< 0.02	5.00	1.89 - 13.25	< 0.002

FC, fold change; RCB, residual cancer burden; HR, Hazard ratio; CI, confidence interval; n.s., not significant.

* only variables significantly associated in the univariate analysis were included in the multivariate analysis.

## DISCUSSION

Despite initial excellent response rates to standard treatment, recurrent disease is still the major cause of mortality in EOC. We demonstrate that, although chemotherapy eliminates most malignant cells, CSCs defined by high ALDH1 activity are left behind in tumors with limited chemotherapeutic response and poor prognosis indicating their pronounced ability to re-initiate cancer.

It is generally accepted that progenitor cells are present in the tumor mass of EOC [26, 27]. In the stochastic model of cancer development treatment failure results from the clonal selection of tumor cells, which acquire genetic and epigenetic alterations during therapy consequently reducing their sensitivity to antineoplastic drugs. In contrast, the CSC hypothesis suggests that a small fraction of cells is intrinsically resistant to chemotherapy and gives rise to tumor recurrence due to preferential proliferation. Consistent with this theory and according to previous publications, we isolated cancerous subpopulations of cells phenotypically resembling CSCs [11, 13, 18]. To enrich for tumor cells we utilized the antigen EpCAM, which has also been observed to be upregulated on CSCs and appeared itself to be an indicator of stem-like features [28]. The combinational use of EpCAM and other CSC-related markers has been found to improve the detection of stem-like cells [29]. Accordingly, ovarian stem-like cells have been characterized previously by their concomitant expression of EpCAM and ALDH1, CD24, CD117 or CD133 [11, 16, 18]. We distinguished tumorigenic from non-tumorigenic cells by their CSC-antigen expression levels. Consistent with published reports we found low- and high-expressing cancer cells with regard to ALDH1 [15, 16] and CD24 [30]; concerning CD117 [31] and CD133 [13] only antigen-positive and -negative cancer cells were observed. Our expression data indicated that ALDH1, CD24 and CD117 are more robust markers of stemness than CD133 since they appeared to be informative in a higher number of tumors. Likewise, a recent report considered CD133 only useful in a minority of EOC patients [22]. Additionally, the application of antibodies targeting different CD133 epitopes was shown to result in distinct expression patterns assigning the sparsest staining to the clone AC133, which was used in our study [32].

The expression variability across individual CSC antigens in our study was in accordance with other reports suggesting that each marker detects a unique rather than the same CSC population [33]. Cancerous ALDH1 expression was the only marker in our study that convincingly predicted the response to chemotherapy and independently prognosticated disease outcome. Also in breast cancer, ALDH1 has been proposed to be the best marker of stemness since fewer cells with this phenotype were required to engraft the disease in mice [34]. ALDH1 is the predominant isoform of a family of cytosolic enzymes that catalyze the oxidation of intracellular aldehydes. It has been suggested to play a drug-metabolizing role possibly protecting CSCs against chemotherapy [35]. In our analysis, we used ALDH1 protein levels, which have been shown to be positively associated with the enzyme activity [11]. An enrichment of tumorous expression of ALDH1 after NAC was associated with a poor chemotherapeutic response. Moreover, the accumulation of ALDH1^high^ CSCs correlated with platinum resistance. Thus, ALDH1 appears to play a significant role in platinum sensitivity, which is vital for the prognosis of EOC patients. In line with these results, carboplatin was shown to enrich ALDH1^+^ CSCs in residual tumors in a xenograft mouse model of EOC [36]. Additionally, several authors have reported an ALDH1 resistance to taxane and platinum *in vitro* [11-13, 15].

According to these data of treatment resistance, ALDH1 served also as a prognostic marker associated with poor clinical outcome. Multivariate analysis identified the accumulation of ALDH1^high^ CSCs in the course of treatment as an independent prognostic factor for OS but not for RFS. ALDH1 staining intensity has been found to be predictive of response to treatment, which might affect not only initial but also relapse therapy. Thus, patients with low counts of ALDH1^high^ cells benefit from each cycle of chemotherapy and consequently exhibit a higher cumulative survival. Alternatively, the difference might be due to non-tumor-related factors influencing survival or to a more accurate assessment of death compared to that of recurrence. Also other authors have associated a high percentage of ALDH1^+^ EOC cells in non-treated patients with short survival times [11-13, 15, 16]. However, our results show for the first time that NAC in EOC selects for ALDH1^high^ CSCs, which intensifies the association of CSCs with poor survival. An increase of ALDH1 expression after NAC identifies tumors with intrinsically aggressive phenotypes. Consistent to our data, ALDH1^+^ cells after NAC in breast cancer but not ALDH1 expression at initial diagnosis influenced prognosis [37].

In advanced EOC, NAC followed by IDS results in comparable HRs for death and progressive disease compared to primary cytoreductive surgery [38]. Benefits of NAC include lower rates of perioperative morbidity, early identification of NRs and evaluation of chemotherapy-induced bystander immune effects [39, 40]. However, critics have expressed concern that NAC, unlike primary surgical treatment, may select for chemoresistant CSCs. Indeed, our results indicated an enrichment of CSCs after NAC. We showed, however, that only a subpopulation of patients is affected. Thus, the analysis of cancerous ALDH1 expression at the time of IDS may identify women, who are candidates for extended treatment including CSC-targeted agents. For instance, Metformin has been shown to be efficient in inhibiting growth and proliferation of ALDH1^+^ CSCs both *in vitro* and *in vivo* [41]. Additionally, clinical trials for the ALDH inhibitor disulfiram have been initiated [42]. Also the pretreatment with gold nanoparticles was found to be promising to reduce acquired platinum-associated stem-like properties [43].

A potential limitation of our study was the use of different tissue types for comparison of chemo-naïve with NAC-treated CSCs. Different findings indicate that signals of the microenvironment are able to modulate the profile of CSCs [5]. However, one recent study demonstrated that frequencies of equally defined CSCs in the primary tumor and intraperitoneal metastases are comparable suggesting amount and characteristics are intrinsic properties [44]. Another restriction of our findings is the lack of experimental validation of stem-like cell behavior. However, other authors have convincingly shown that ALDH1^+^ EOC cells have the ability to engraft in immunodeficient mice and when propagated recapitulate their original tumor phenotype [11-13, 15, 16].

In conclusion, the key contribution of our analysis is to better understand the evolution of resistance mechanisms under the selective pressure of chemotherapy *in vivo*. For patients with high amounts of ALDH1^high^ CSCs after NAC our study demonstrated an elevated risk of poor outcome. Thus, we suggest ALDH1 to be a pivotal marker in EOC that may improve the accuracy of clinical outcome predictions and the choice of appropriate treatment.

## MATERIALS AND METHODS

### Patients and specimen

The study population consisted of a retrospective sample of 55 patients with advanced EOC (serous-papillary, FIGO stage IIIC/IV) diagnosed at the University of Bonn between 2002 and 2012. Patients were treated with neoadjuvant chemotherapy under clinical trial conditions [2] or because they were considered to be poor candidates for upfront primary cytoreductive surgery [45]. Clinical information was obtained from medical records; follow-up data were updated until July 2014. After approval of the Institutional Review Board the following matched pairs of histological sections were selected for morphological and immunohistochemical analysis: tumor tissue obtained at the time of initial diagnosis by laparoscopy from therapy-naïve patients, tumor tissue collected at the time of IDS after NAC. Histopathological diagnosis was determined based on World Health Organization (WHO) criteria, tumor grade on Gynecologic Oncology Group (GOG) criteria. The International Federation of Gynecology and Obstetrics (FIGO) system was used to assign the tumor stage. According to the National Comprehensive Cancer Network (NCCN) guidelines residual disease < 1cm defines optimal cytoreduction [45]. In Europe, since 2010 the term ‘optimal cytoreduction’ is reserved for women with no macroscopic residual disease (NRD) [46]. In our study, we favored NCCN guidelines due to the high number of women (44.80%) treated in the years before 2010. Patient baseline characteristics are listed in Table 1.

### Assessment of chemotherapy response

Histopathological criteria are considered the gold standard to differentiate between chemotherapeutic Rs and NRs. Therefore, the morphological response to chemotherapy was assessed by an extensive evaluation of all specimens taken at the time of IDS [47, 48]. According to published criteria, samples with no residual tumor or marked signs of tumor regression were categorized as histopathological Rs; samples with no signs of tumor regression or minimal regressive changes in < 50% of tumor cells were classified as NRs [39, 49]. Additionally, relapse-free intervals were determined for the analysis of treatment response to platinum-containing regimen. Disease that recurred within 6 months of completing chemotherapy was defined platinum-resistant; disease that relapsed within 6-12 months was considered intermediate and disease that relapsed after a 12-month interval was considered highly sensitive to platinum [50].

### Immunohistochemistry

Areas of EOC were identified in sections stained with hematoxylin and eosin. IHC of ALDH1, CD24 [51] and CD117 was performed on 2-3μm formalin-fixed paraffin-embedded tissue specimens using an automated staining system (Medac 480 S Autostainer; Medac, Wedel, Germany). The reaction was developed with HRP-conjugated goat anti-mouse/rabbit/rat IgG and the DAB system (Medac). CD133 IHC was carried out on the Ventana BenchMark Ultra (Roche, Basel, Switzerland) using Ventana OptiView reagents and the DAB detection kit. Supplementary Table 1 reports the antibodies used for IHC.

### Evaluation of immunoreactions

Immunostained cells were analyzed with a Leica DM LB2 microscope (Leica Microsystems Wetzlar GmbH, Wetzlar, Germany). To minimize interobserver variability all specimens were analyzed by the same person (B.R.) in a blinded fashion. ALDH1 was considered positive when the cytoplasm showed a positive reaction [11]; for CD24 total staining was determined [51]; CD117 was scored as positive if the staining was localized to the cell membrane and cytoplasm simultaneously [52]; for CD133 cell membrane labeling was regarded positive [53]. Tumoral expression of ALDH1, CD24, CD117 and CD133 was assessed using a semiquantitative immunoreactivity score ranging from 0 (negative) to 12 (strongly positive), calculated as the product of staining intensity (0 = no staining; 1 = weak staining; 2 = moderate staining; 3 = intense staining) and staining area (0 = 0%; 1 = < 10%; 2 = 11-50%; 3 = 51-80%; 4 = 81-100%). To account for different quantities of viable tumor in non- and NAC-treated tissue samples the amount of vital tumor was morphologically determined and clustered as follows: 100%, > 50%, < 50%, 0% of vital tumor. For statistical analysis these quantities were set to 1, 0.75, 0.25 and 0.05 representing the mean of the intervals and accounting for non-observed vital cells in the last group. Expressions of stemness antigens are given as semiquantitative immunoreactivity scores normalized by the amount of vital tumor (relative immunoreactivity score, denoted as IRS). ALDH1 staining intensity was also recorded for stromal cells adjacent to the cancer using criteria mentioned above.

### Flow cytometry

Ascites was collected at the time of initial diagnosis in a subset of 15 patients. Primary antibodies are provided in Supplementary Table S1, secondary antibodies in Supplementary Table S2. Data were obtained on a LSR II flow cytometer (BD Biosciences) evaluating at least 100.000 events per sample after excluding debris and doublets. Benign cells expressing CSC-related antigens were excluded from flow cytometric analysis by electronic gating. ALDH1 was stained intracellularly using the Cytofix/Cytoperm kit (BD Biosciences). Analysis was performed by FlowJo software (TreeStar, Olten, Switzerland).

### Statistical analysis

Statistical analysis was carried out using SPSS version 21 (IBM Corp, Armonk, NY, USA) and ‘R’ version 2.15.1 (The R Foundation for Statistical Computing, Vienna, Austria). Fold change (FC) was defined as the ratio of the IRS in pre-NAC samples to that in post-NAC tissue. Due to our inability to enumerate all tumor cells in a patient a pseudocount of one was added to all IRS values to account for unobserved immunopositive cells [54]. The median was used as a cut-off point to assign tumors into high and low ALDH1, CD24, CD117 and CD133 reactivity groups. Comparisons between continuous data were carried out using the Mann-Whitney U and the Kruskal-Wallis test; comparisons between categorical variables were performed using the chi-square test; *p*-values for tests with only a small number of counts were computed based on Monte Carlo simulations using 1.000.000 replicates. Cumulative survival analysis was performed using Kaplan-Meier method; curves were compared with log-rank test. Multivariate survival analysis was performed using the Cox's proportional hazard regression model. The median follow-up time was calculated by reverse Kaplan-Meier estimator [55]. Results with a *p*-value < 0.05 were considered to be significant.

## SUPPLEMENTARY MATERIAL FIGURES AND TABLES


