# Supplementary material for: Accumulation of ALDH1-positive cells after neoadjuvant chemotherapy predicts treatment resistance and prognosticates poor outcome in ovarian cancer
Source: Oncotarget. 2015 May 11;6(18):16437–48. doi: 10.18632/oncotarget.4103 (PMC4599280; doi:10.18632/oncotarget.4103)
Supplement: Supplementary file 1 [file oncotarget-06-16437-s001.pdf]

Accumulation of ALDH1-positive cells after neoadjuvant chemotherapy predicts treatment resistance and prognosticates poor outcome in ovarian cancer

Supplementary Material

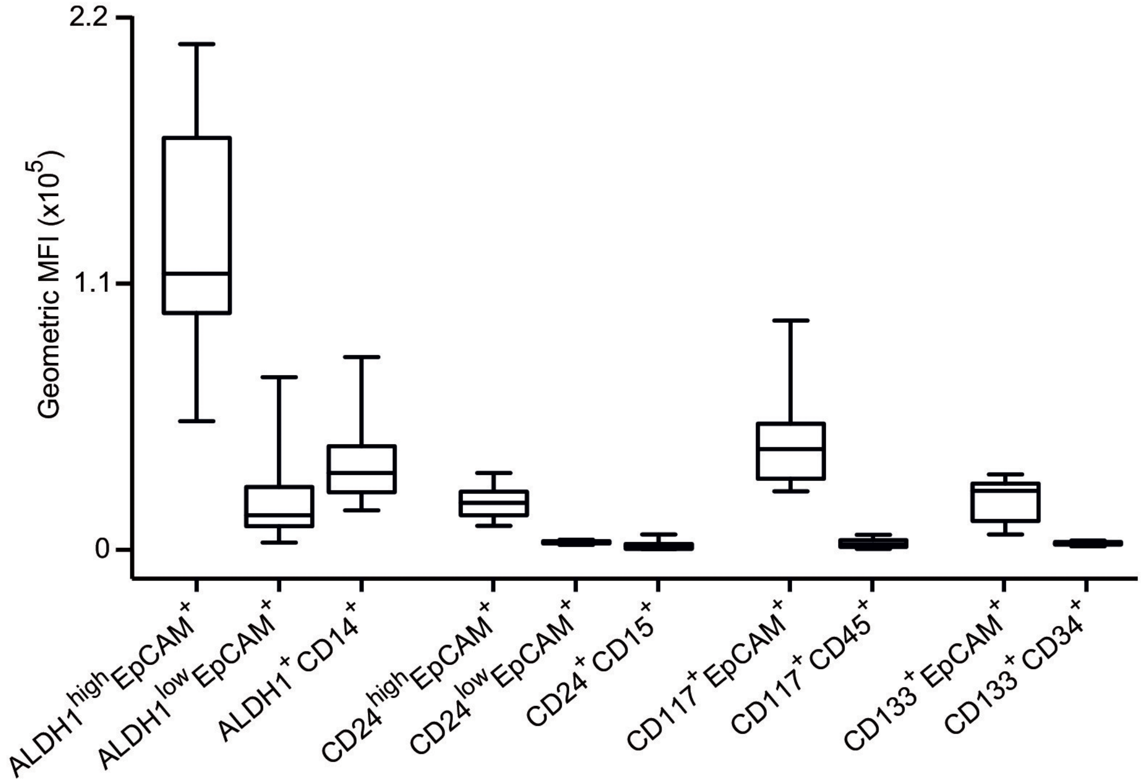

Supplementary Figure S1: Stemness antigens in paired malignant and benign cells. Levels of CSC-related markers were determined in ascitic samples of therapy-naïve patients with EOC. Cytoplasmic ALDH1 and membranous CD24, CD117 and CD133 expression was assessed by flow cytometry on $EpCAM^+$ tumor cells, $CD14^+$ myeloid cells, $CD15^+$ granulocytes, $CD45^+$ immune cells and $CD34^+$ hematopoietic progenitor cells. Data are given as geometric mean fluorescence intensity (MFI) values; bars indicate the median; box plots extend from the 25th to the 75th percentiles; whiskers indicate minimum and maximum values.

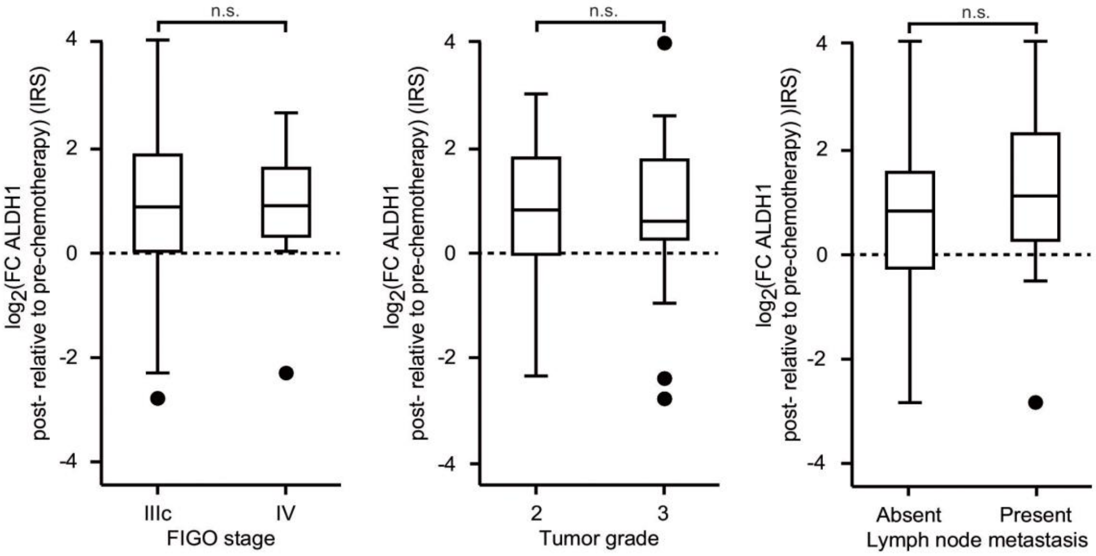

Supplementary Figure S2: Tumoral ALDH1 expression is not correlated with clinical parameters. Expression of ALDH1 was assessed by immunohistochemistry in tissue before and after neoadjuvant chemotherapy (NAC); fold changes (FC) in expression levels were calculated as the ratio of the immunoreactivity scores in pre- to these in post-NAC tissue; classification was undertaken according to FIGO stage, tumor grade and lymph node metastasis. Dashed lines indicate no change of CSC frequency. Box plots summarize the median, 25th and 75th percentiles, the whiskers and outliers (n.s., not significant).

Supplementary Table S1: Antibodies for immunohistochemistry and flow cytometry.

Antigen	Clone	Isotype	Dilution	Source
ALDH1	44/ALDH1	monoclonal mouse IgG1	1:200	BD Biosciences, Heidelberg, Germany
EpCAM	HEA-125	monoclonal mouse IgG1	1:150	Miltenyi Biotec, Bergisch Gladbach, Germany
EpCAM	EBA-1	monoclonal mouse IgG1	1:5	BD Biosciences, Heidelberg, Germany
CD14	M5E2	monoclonal mouse IgG2a	1:100	BD Biosciences, Heidelberg, Germany
CD15	VIMC6	monoclonal mouse IgM	1:100	Miltenyi Biotec, Bergisch Gladbach, Germany
CD19	HIB19	monoclonal mouse IgG1	1:100	BD Biosciences, Heidelberg, Germany
CD24	SWA-11	monoclonal mouse IgG1	1:40	Kindly provided by P. Altevogt, DKFZ, Heidelberg, Germany
CD45	HI30	monoclonal mouse IgG1	1:100	BD Biosciences, Heidelberg, Germany
CD117	A4502	polyclonal rabbit IgG	1:200	Dako, Hamburg, Germany
CD133	AC133	monoclonal mouse IgG1	1:25*, 1:100**	Miltenyi Biotec, Bergisch Gladbach, Germany

*, dilution used in immunohistochemistry.
**, dilution used in flow cytometry.

Supplementary Table S2. Secondary antibodies for flow cytometry.

Name	Isotype	Source
Goat anti-mouse antibody	IgG (H+L)	Life Technologies, Darmstadt, Germany
Goat anti-rabbit antibody	IgG (H+L)	Life Technologies, Darmstadt, Germany
